# Toxic Shock Syndrome Caused by Streptococcus dysgalactiae Subspecies equisimilis: A Report of a Rare Pediatric Case

**DOI:** 10.7759/cureus.78573

**Published:** 2025-02-05

**Authors:** Nao Koizumi, Yoshiaki Shikama, Tadayoshi Ikebe, Hiroyuki Nagafuchi, Tomoyuki Imagawa

**Affiliations:** 1 Infection and Immunology, Kanagawa Children's Medical Center, Yokohama, JPN; 2 Bacteriology, National Institute of Infectious Diseases, Tokyo, JPN; 3 Critical Care Medicine, Kanagawa Children’s Medical Center, Yokohama, JPN

**Keywords:** child case, japan, stc1400, streptococcal toxic shock syndrome, streptococcus dysgalactiae subsp. equisimilis

## Abstract

Streptococcal toxic shock syndrome (STSS) is a severe invasive infection that has a high mortality rate. It is mainly caused by *Streptococcus pyogenes*. Nevertheless, STSS is also known to be caused by a group G *Streptococcus* identified as *Streptococcus dysgalactiae* subsp. *equisimilis* (SDSE). Invasive SDSE infection predominantly occurs in elderly individuals but is extremely rare in children. Furthermore, reports on STSS caused by SDSE are limited; hence, the clinical aspects and type of invasive mature M protein (*emm*) gene involved are unknown. A five-year-old girl with Down syndrome, tracheomalacia, and laryngomalacia presented to the emergency department with generalized edema of her face and upper body. She had undergone a tracheostomy and was receiving home ventilation. On admission, she showed hypotension (64/22 mmHg) with impaired consciousness. Blood testing showed elevated levels of inflammatory markers. Blood culture revealed the presence of SDSE and she was consequently diagnosed with STSS. The patient responded well to treatment and was discharged three weeks after admission. The *emm *subtype of the isolated SDSE strain was *stC1400,* with mutations in *csrS *and streptolysin S regulatory RNA in group G *Streptococcus* (*srrG*) genes. In this article, we report a case of STSS in a child caused by the *stC1400*strainof SDSE.

## Introduction

Streptococcal toxic shock syndrome (STSS) is diagnosed based on rapidly progressing symptoms, including necrotizing fasciitis, and signs of shock, hypotension, renal impairment, coagulopathy, liver involvement, and acute respiratory distress syndrome. This syndrome is associated with a high mortality rate. *Streptococcus pyogenes*, a group A *Streptococcus* (GAS), is recognized as the most common causative pathogen in patients with STSS. Nonetheless, severe invasive streptococcal infection, including STSS, may also be caused by group G streptococci, particularly *Streptococcus dysgalactiae *subsp. *equisimilis* (SDSE) [1-3]. This is phylogenetically similar to *Streptococcus pyogenes*, which explains the overlapping spectrum of infections caused by these species [4]. Currently, the number of reports on STSS caused by SDSE is limited, and the clinical aspects and type of invasive M protein (*emm*) gene involved are unknown. Invasive SDSE infections are mainly detected in elderly patients with comorbidities such as diabetes mellitus, cardiovascular diseases, immunosuppression, and cancer, and they are rarely detected in pediatric patients [5,6]. In this article, we report the first case of STSS in a child caused by SDSE with an *emm *type *stC1400* gene.

## Case presentation

The patient was a five-year-old girl diagnosed with Down syndrome. She presented with developmental delay, pulmonary hypertension, atrial septal defect, and coloboma. She had undergone surgery for a congenital diaphragmatic hernia at 10 days of age and experienced recurrent respiratory infections during her neonatal and toddler periods. At the age of three, she underwent a gastrostomy due to difficulty with oral intake. Subsequently, due to tracheomalacia and laryngomalacia, she underwent a tracheostomy and was receiving home ventilation. The patient presented to the emergency department with generalized edema of her face and upper body. She had been on a medical short stay for one week before visiting our hospital. Three days before the visit, she developed a fever that subsided without medication. On the day she left the short-stay unit, her mother noticed edema and brought her to our hospital for examination.

**Figure 1:**
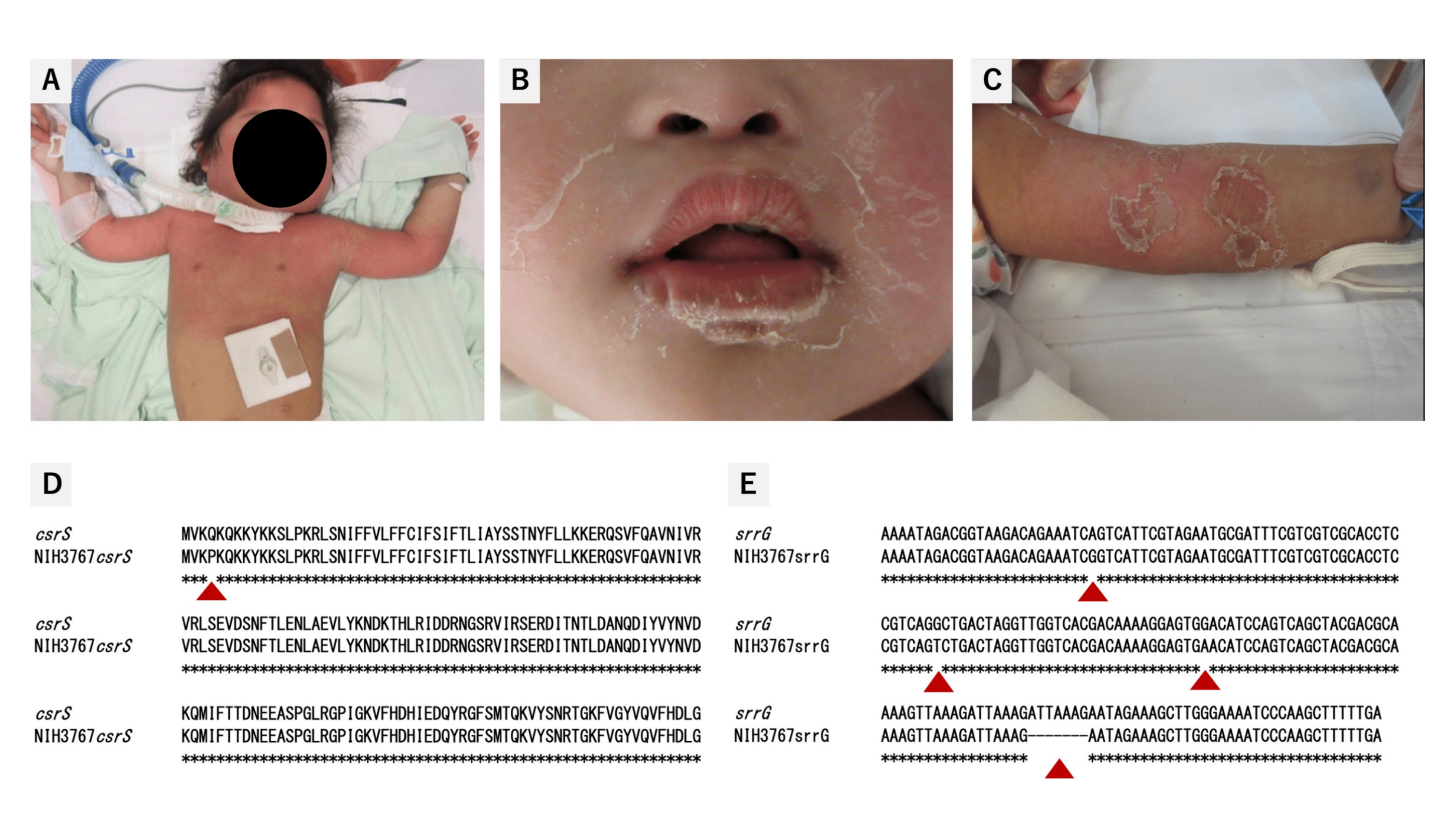
Clinical photographs and polymerase chain reaction (PCR) analysis of csrS and srrG genes. On admission, erythema was observed on the head, neck, and upper body (Panel A). Desquamation was noted on the lips (Panel B), which spread from the upper arms to the trunk (Panel C). The isolate had a proline–glutamine substitution in the *csrS* gene (Panel D), and deletion of ATTAAAG and three other mutations in the *srrG* gene (Panel E). These alterations resulted in negative regulation of the *sagA *gene. *sagA*: streptolysin S-associated; *srrG*: streptolysin S regulatory RNA in group G *Streptococcus*.

On admission, the patient was afebrile (35.9℃) but had hypotension (64/22 mmHg) along with impaired consciousness. Erythema with edema was observed on her face, upper limbs, and the upper part of the trunk. Desquamation appeared on the lips and spread from the upper part of the trunk (Figure 1). She did not have symptoms of influenza-like syndrome, such as fever, chills, myalgia, nausea, vomiting, and diarrhea. Based on the clinical findings, she appeared to develop anaphylactic shock and was promptly admitted to the pediatric intensive care unit. Despite repeated administration of epinephrine and fluid challenges, she remained hypotensive. Blood testing conducted in the emergency department revealed mild anemia (hemoglobin: 96 g/L), hyponatremia (sodium: 116 mEq/L), hyperkalemia (potassium: 5.4 mEq/L), kidney injury (creatinine: 7 mg/L), and elevated levels of inflammatory markers (C-reactive protein: 152 mg/L) (Table 1). Based on the clinical information, she was diagnosed with septic shock and received an infusion of piperacillin-tazobactam (350 mg/kg/day). After initiating antibiotic treatment, the blood pressure gradually stabilized within normal range.

**Table 1:** Results of blood tests on admission and days four and five.

Blood variables	Result			Reference range
	Admission	Day 4	Day 5	
White blood cell count (×10^9^/L)	7.3	18.9	10.3	5.5-15.5
Hemoglobin (g/L)	96	81	92	115-155
Blood platelets (×10^9^/L)	115	79	54	150-400
Total protein (g/L)	45	64	64	61-79
Aspartate aminotransferase (U/L)	30	51	36	13-30
Alanine aminotransferase (U/L)	17	25	20	5-45
γ-glutamyl transpeptidase (U/L)	5	7	9	13-64
Total bilirubin (mg/L)	2	4	5	4-12
Prothrombin time-international normalized ratio	2.12	1.23	1.21	0.90-1.10
Fibrinogen (mg/L)	3960	2780	2510	2000-4000
D-dimer (µg/L)	2650	4840	5800	<1000
Blood urea nitrogen (mg/L)	84	73	75	50-180
Creatinine (mg/L)	7	3.8	3.6	3-7
Sodium (mmol/L)	116	135	141	138-145
Potassium (mmol/L)	5.4	3.7	3.4	3.5-5.0
Chlorine (mmol/L)	84	100	106	98-106
C-reactive protein (mg/L)	152	208.7	173	0.0-1.8

The day after admission, aerobic and anaerobic blood cultures based on samples collected at admission revealed the presence of Gram-positive streptococci. Considering the clinical features, laboratory data, and isolation of streptococci from a sterile site, the patient was diagnosed with STSS. The regimen for empiric therapy was changed to ampicillin (200 mg/kg/day) and clindamycin (40 mg/kg/day) with intravenous immunoglobulin (1 g/kg on day one, followed by 0.5 g/kg on days two and three). The initial bacterial identification of the isolates and antimicrobial susceptibility testing for a minimum inhibitory concentration were performed using a VITEK 2 system (bioMérieux, Marcy-l'Étoile, France). Three days after admission, SDSE was identified. The isolates were sensitive to penicillin G (≤0.06 μg/mL), amoxicillin (≤0.25 μg/mL), ceftriaxone (≤0.12 μg/mL), and vancomycin (0.5 μg/mL), whereas they were resistant to erythromycin (8 μg/mL) and clindamycin (<0.25 μg/mL).

Blood cultures from samples obtained at admission and day one yielded positive results; consequently, we performed enhanced computed tomography. There was no abscess formation or evidence of the source of bacteremia. Because the first blood culture yielded negative results, we administered clindamycin and ampicillin for seven and 14 days, respectively. On day four of antibiotic administration, levels of inflammation markers peaked (C-reactive protein: 208.7 mg/L) and the patient experienced transient anemia and thrombocytopenia (hemoglobin: 81 g/L; platelets: 79× 10^9^/L) requiring transfusion (Table 1). As blood data improved, erythema and desquamation developed into purpura or hyperpigmentation (Figure 1). On day 15 after hospital admission, the patient was moved to the general ward; she was subsequently discharged 21 days after admission.

The strain of SDSE isolated from the patient's blood culture was submitted to the National Institute of Infectious Diseases (NIID) for analysis of the *emm* type, which encodes the M protein associated with pathogenicity. The *emm* typing, based on polymerase chain reaction (PCR), involved direct sequencing the amplified DNA fragments and utilizing the Centers for Disease Control and Prevention (CDC) *emm* sequence database for *emm* type identification. Genetic testing revealed that the *emm* type of the isolated SDSE was *stC1400*. Further analysis was conducted on the pathogenicity of SDSE with the *emm* type of *stC1400*, and *csrS* and streptolysin S regulatory RNA in group G *Streptococcus* (*srrG*) mutations were observed in the isolated SDSE (Figure 1).

## Discussion

This case is the third instance of STSS caused by SDSE found in a pediatric case. According to the CDC guidelines, patients meeting the clinical case definition of a sudden onset of shock, organ failure, and isolation of GAS from a normally sterile site should be diagnosed with STSS [7]. Since the late 1980s, STSS caused by *Streptococcus pyogenes* (GAS) has been considered a serious health concern in numerous countries. In 2022, a historic increase in pediatric invasive group A streptococcal infections was reported globally [8-10]. Despite medical progress in the care of patients with septic shock, this condition remains associated with a high mortality rate (38%) [11]. STSS is mainly caused by *Streptococcus pyogenes* and, more rarely, SDSE [12]. Of all cases of STSS reported in Japan, 61.9% are caused by GAS, with group G *Streptococcus* being rare at 20.9% [13]. SDSE expresses Lancefield group C or G antigens and exhibits strong β-hemolysis. Similar to group A streptococci, SDSE possesses virulence factors, including M protein, streptolysin O, streptolysin S, streptokinase, hyaluronidase, and C5a peptidase, among others. This *Streptococcus* is considered a component of the normal human flora, typically residing on the skin and within the oropharynx, gastrointestinal tract, and urogenital tract. Invasive SDSE infection primarily occurs in the elderly who have an underlying malignancy (e.g., cardiac disease or diabetes) [14], while it rarely occurs in children. Takahashi et al. identified the median age of patients with SDSE diseases as 75 years (range: 19-103 years), indicating that the patients infected with SDSE were significantly older than those infected with GAS (p < 0.01) [5]. Thus far, only two pediatric cases of STSS caused by SDSE have been reported worldwide. Yamaoka et al. reported a case of STSS caused by SDSE that developed within 12 hours of birth [15], while Rodríguez-Muñoz et al. reported a case in a previously healthy three-year-old in Mexico [16]. It is generally considered that newborns have low immunity. Down syndrome is associated with immune abnormalities, making individuals more susceptible to respiratory infections, type 1 diabetes, alopecia areata, vitiligo, celiac disease, and other autoimmune diseases. However, not all individuals with Down syndrome develop these conditions despite the increased risk. This may be due to the unique characteristics of causative pathogens within group G streptococci.

In the present case, the NIID conducted genetic testing and discovered mutations in *csrS* and *srrG* genes in the isolated strain (Figure 1). The *csrS* gene in GAS negatively regulates the expression of streptolysin O, an important toxic factor in neutrophil necrosis. In addition, in the case of SDSE, the mutation in the *csrS* gene is associated with the virulence of the bacterium. In *emm* types *stG6792* and *ST17*, Ikebe et al. reported that mutations in *csrS/csrR* were found in 19.0% of patients with STSS; in contrast, mutations were not found in non-invasive SDSE isolates [17]. The *srrG* gene mutation is a natural mutation in the regulatory gene that can increase the invasiveness and virulence of SDSE strains. Mutations of *srrG* were identified in STSS-SDSE strains and found to be associated with elevated expression of the streptolysin S gene cluster and enhanced pathogenicity in mice. The *srrG* mutation enhances the expression of the streptolysin S-associated (*sagA*) gene and increases mouse virulence. The product of the *sagA* gene is modified and exported as the cytotoxin, streptolysin S, a virulence factor that inhibits the response of neutrophils. The *emm* type of the isolated strain in this case was *stC1400*. However, the clinical course of invasive SDSE infections with *emm* type *stC1400* has been unclear due to the limited number of cases. We found four articles published in PubMed up to December 03, 2024. Rojo-Bezares et al. reported that *emm* type *stC1400* was frequently found in invasive SDSE infections [18]. However, to date, no reports exist regarding mutations in toxic genes. Our case report describes, for the first time, how SDSE with *emm* type *stC1400* can have *csrS/srrG* gene mutations in humans, supporting the previously reported hypothesis that *emm* type *stC1400* is an invasive genotype. Given the limited number of reported cases, conducting a comprehensive clinical review is challenging. Therefore, we propose expanding genomic surveillance to monitor the prevalence of *stC1400* and associated mutations across different regions. Longitudinal studies will be essential to correlate genetic profiles with disease progression and outcomes, while collaborations with global pathogen surveillance networks will help identify shared genetic markers. These initiatives will provide valuable insights into the pathogenic mechanisms of SDSE and inform the development of improved diagnostic, therapeutic, and preventive strategies.

However, STSS caused by SDSE is known to have a better prognosis compared to that caused by GAS (38%) [11] and the present case responded well to treatment. From 2014 to 2016, 173 SDSE strains were reported in Japanese patients with STSS, with a mortality rate of 31.2%. An insufficient white blood cell response (<5,000 cells/µL) and thrombocytopenia on admission were associated with a significantly higher risk of poor outcome, with a respective odds ratio of 3.6 (95% CI: 1.2-11.5; p = 0.04) and 4.5 (95% CI: 1.6-12.2; p <0.01). These parameters were identified as poor prognostic factors for invasive SDSE infections [5]. Although the present patient was at risk of a poor outcome due to thrombocytopenia, our case eventually showed a relatively favorable course of treatment. Macrolide- and clindamycin-resistant streptococci have been reported in several countries. In line with our case, Prabu and Menon have shown a strong association between *stC1400* and resistance to erythromycin in their study [19]. Linezolid is a promising alternative adjunctive agent. However, clinical evidence supporting the use of linezolid as an antitoxin agent in severe GAS infections is currently insufficient [20]. In our case, we did not change clindamycin to linezolid since the patient responded well to the former. Therefore, even in pediatric cases, the same antibiotics are recommended as those used for elderly patients with STSS caused by SDSE.

## Conclusions

We experienced a case of severe streptococcal infection in a child caused by SDSE *emm* type *stC1400*. Group G streptococcal infections are more common in elderly individuals with complications but rarely occur in children. Furthermore, the pathogenicity of this *emm* type is seldom reported in the literature, and it is unknown whether the prognosis of SDSE infection with *emm* type *stC1400* is poor or not. Based on our case and a literature review, we suggest that this *emm* type may be associated with virulence gene mutations.
